# Influence of water availability on gender determination of gametophytes in a diploid–polyploid complex of a xerophytic fern genus

**DOI:** 10.1093/aobpla/plv047

**Published:** 2015-05-02

**Authors:** Santiago Pajarón, Emilia Pangua, Luis G. Quintanilla, Ares Jiménez

**Affiliations:** 1 Departamento de Biología Vegetal I. Facultad de Biología, Universidad Complutense, C/José Antonio Novais 2, 28040 Madrid, Spain; 2 Departamento de Biología y Geología, Física y Química Inorgánica, Escuela Superior de Ciencias Experimentales y Tecnología, Universidad Rey Juan Carlos, C/Tulipán s/n, 28933 Móstoles, Madrid, Spain

**Keywords:** Allopolyploid, antheridiogen, *Cheilanthes*, environmental sex determination, gametophyte gender, mixed mating, water availability, xerophytic ferns

## Abstract

Environmental sex determination (ESD) is present in several animal and plant lineages. Diverse factors such as temperature, light or water availability have been described as sex determinants in these organisms. Among plants, ferns frequently display ESD. This work compares the effect of different levels of water availability in two diploid species of the xerophytic fern genus *Cheilanthes* and in their derived tetraploid, and if they are sensitive to antheridiogen (i.e. maleness-inducing pheromone). Different watering regimes were applied to isolated gametophyte cultures of the three study species. Gametophyte survival, size, gender and sporophyte production were assessed after 13, 18 and 23 weeks of culture. Cultures combining spores and adult gametophytes were established to test the effect of antheridiogen. Isolated gametophytes had an asexual to female to bisexual sequence that did not depend upon the degree of soil moisture. Both gender expression and growth reduction in response to water scarcity of the allotetraploid were more similar to those of one of the diploid parents. In all watering regimes, survival was higher in the allotetraploid, suggesting hybrid vigour, whereas automixis rate was similar in the three species and reached ∼50 % at high moisture. This breeding system can ensure reproduction in the absence of males. In the three species, female gametophytes produced antheridiogens that enhanced maleness. This promotes a mixed mating system that could be favourable for ferns growing in xeric habitats.

## Abstract (precis)

Environmental sex determination is present in several animal and plant lineages, in which gender depends on diverse factors such as temperature, light and water availability. This study examines effects of water availability and pheromones on the production of female and male organs by three fern species adapted to dry habitats. Isolated individuals become first female and then bisexual, irrespective of the degree of soil moisture, and, consequently, may self-fertilize. However, female individuals release pheromones that induce maleness in nearby individuals, thus favouring cross-fertilization.

## Introduction

Sexual reproduction provides organisms with an opportunity to exchange genetic information with their conspecifics, which potentially increases genetic diversity and, with it, the ability of species to adapt to changing environmental conditions. Sexual reproduction is widespread throughout the tree of life, even though the mechanisms that dictate which individuals reproduce as males, females or bisexuals are diverse. These mechanisms fall into two main categories, namely genetic sex determination (GSD), whereby sex is fixed by the genotype; and environmental sex determination (ESD), whereby sex is determined by environmental factors. In most animal species, in particular mammals and birds, GSD is the rule (Wedekind and Stelkens 2010), although ESD is present in some lineages, such as reptiles (e.g. Pieau *et al*. 1994), fishes (e.g. Ospina-Álvarez and Piferrer 2008), nematodes and echiurid worms (Bacci 1965). Additionally, in some other reptiles and fish species, sex is affected by a combination of both genetic and environmental factors (Valenzuela *et al*. 2003; Sarre *et al*. 2004; Baroiller *et al*. 2009). Moreover, there are some animal and plant species that are able to change sex expression in response to environmental changes (Freeman *et al*. 1980; Policansky 1982; Stelkens and Wedekind 2010).

Whereas most animals are unisexual, the sex expression of plants shows a large diversity, from dioecious species, where individuals can be male or female, to monoecious species, whose individuals can have both male and female flowers or perfect (i.e. hermaphroditic) flowers (Ainsworth 1999). Among land plants only ∼10 % are dioecious, and this sexual system is distributed in such different groups as liverworts, mosses, gymnosperms and angiosperms (Ming *et al*. 2011). Dioecy has been hypothesized to derive from hermaphroditism and this shift could be due to changes in sex gene expression or to the influence of ESD (Barrett 2002; Talamali *et al*. 2003; Charlesworth and Mank 2010). The main selective factors influencing this process have been suggested to be the avoidance of inbreeding and resource allocation at the time of reproduction (Charlesworth and Guttman 1999).

Temperature, photoperiod, light availability, nutrient concentration, pH, water availability, trauma and parasite effects, among others, have been described as major determinants of sex in organisms subject to ESD (Freeman *et al*. 1980; Korpelainen 1990, 1998, and references therein). A general trend in these organisms is that individuals under conditions favourable to growth (e.g. high light, water and nutrient availability) tend to develop as females, whereas individuals under unfavourable conditions (e.g. limited growth resources, parasites, physical injuries, dry soils and high temperatures) tend to develop as males (Sarkissian *et al*. 2001). Consequently, barring some exceptions like mosses, which tend to femaleness under harsh conditions (Stark *et al*. 2005), males tend to be over-represented in stressful environments (Pannell 1997).

Some land plant species exhibiting GSD have heteromorphic (i.e. *X* and *Y*, or *W* and *Z*) sex chromosomes. For example, there is evidence of sex chromosomes in several mosses and liverworts, but there is no evidence of sex chromosomes or GSD in ferns (Ming *et al*. 2011). Many ferns apparently rely on ESD. Unlike the heterosporous flowering plants, all but a small number of ferns are homosporous and produce spores of similar size with the potential to develop into male, female or bisexual gametophytes, although their default sexual ontogeny typically leads to female or bisexual states (Jesson *et al*. 2011; Sánchez Vilas and Pannell 2012). In many species, unfertilized female gametophytes can develop male sex organs and become bisexual, potentially a way to assure sexual reproduction (Warner 1988; Verma 2003). Antheridiogen pheromones, which are present in many homosporous fern species (Yamane 1998; Schneller 2008), are acknowledged to be the main ESD factor in ferns. Antheridiogens are gibberellin-related molecules which are released by archegoniate (i.e. female or bisexual) gametophytes. Antheridiogens can induce spore germination in the dark and the formation of antheridia on sexually immature gametophytes (Döpp 1950; Tanurdzic and Banks 2004; Quintanilla *et al*. 2007; Jiménez *et al*. 2008). The influence of other environmental factors, such as light availability, nutrient availability, culture density or presence of sucrose in the culture media, has received some attention. An increase in photon irradiance can modify the sex ratio in *Equisetum* gametophytes favouring development of females (Guillon and Fievet 2003), while the presence of sugar in the culture media favoured the development of males (Guillon and Raquin 2002). Limited nutrient supply favoured maleness in *Woodwardia* (DeSoto *et al*. 2008), as well as high culture density that increased males and asexuals in *Osmunda* (Huang *et al*. 2004).

Polyploidy, i.e. multiplication of the diploid chromosome complement, is considered an important mode of speciation in plants. Polyploidization triggers changes of various kinds (e.g. metabolic, physiologic and gene regulation) with respect to the diploid parents and these changes may provide polyploids with novel abilities to face environmental requirements (Levin 1983; Wendel 2000). Polyploidization can also influence reproduction. In mosses, for example, it has been suggested that polyploidy would allow for the existence of gametophytes with both *X* and *Y* chromosomes, and these bisexual gametophytes may exhibit improved environmental tolerances relative to single-sex gametophytes (Lewis 1961; Wyatt and Anderson 1984). There is empirical evidence that polyploidy affects the frequency of inbreeding vs. outcrossing (Husband *et al*. 2008). Moreover, it is widely held that polyploids have higher selfing rates than their diploid relatives (Stebbins 1950; Soltis and Soltis 2000; Mable 2004; Barringer 2007). Regardless, despite the relevance of the effect of polyploidy in the breeding system, its influence on the patterns of sexual maturation under ESD has been barely explored.

Allopolyploidy, i.e. hybridization followed by chromosome doubling, is a frequent mode of secondary speciation in vascular plants (Leitch and Bennett 1997; Haufler 2008). The occurrence of diploids and their derived polyploids in the same area provides an excellent natural experiment to test the unique environmental responses that may exist across ploidy levels. Life-history traits of an allopolyploid can resemble one of the parents, be intermediate or even fall outside the range of variation exhibited by both parents (Sessa and Givnish 2014). The latter option may favour niche divergence and thus coexistence of different cytotypes (Rieseberg *et al*. 1999). In addition, allopolyploids may have hybrid vigour, i.e. higher fitness as a consequence of increased heterozygosity (Comai 2000). Despite the importance of allopolyploidy in many plant lineages, its phenotypic consequences remains perhaps one of the most neglected areas of research in plant evolutionary biology.

In this study, we focussed on a complex of two diploids ferns and their derived allotetraploid that largely overlap in their distribution and live in similar habitats, namely rocky slopes facing south in more or less xeric conditions. Thus, this group provides a model to test the effect of water availability on sexual development, and the different responses of related diploids and tetraploids. The specific questions that we aimed to address were: (i) does low water availability cause reduced growth and favour maleness? (ii) Do diploids and allotetraploids show different vegetative and sexual responses to varying levels of water availability? (iii) Do the reproductive behaviours of the three species under different moisture levels represent advantages in their xeric habitats? (iv) Is there an antheridiogen system that affects sex expression of this species group?

## Methods

### Study species

Three *Cheilanthes* (Pteridaceae) species were studied: the allotetraploid (2*n* = 4*x* = 120) *C. tinaei* and its diploid (2*n* = 2*x* = 60) parents: *C. hispanica* and *C. maderensis. Cheilanthes tinaei* and *C. maderensis* are both located in the western Mediterranean basin and in some Macaronesian archipelagos whereas *C. hispanica* is subendemic to the Iberian Peninsula, with some populations also in Algeria and southern France. All three species grow in crevices of exposed siliceous outcrops from 0 to 1350 m above sea level (Garmendia 1986) and can be occasionally found in mixed populations. However, they tend to occupy different microhabitats, with an increase of water requirements in the order: *C. hispanica* (most xerophytic) < *C. tinaei* < *C. maderensis* (Garmendia 1986; Amor *et al*. 1993). The three species are hemicryptophytes and have rhizomes that may branch but do not display extensive clonal growth. The leaves, which form dense apical rosettes, are bigger in *C. tinaei* (up to 30 cm long) than in *C. hispanica* (<26 cm) and *C. maderensis* (<18 cm) (Jermy and Paul 1993). Mature gametophytes develop a typical heart-shape and may bear female (archegonia) and/or male (antheridia) sex organs, and they can be males, females or bisexuals.

### Plant material

Spores of the three species were obtained from one population per species, 15 individuals per population, in central Spain: *C. hispanica* in El Relumbrar range (Albacete province, 38°40′N, 2°38′W), *C. maderensis* in La Fregeneda (Salamanca province, 41°01′N, 6°55′W) and *C. tinaei* in Torrelodones (Madrid province, 40°35′N, 3°56′W). These localities followed the same relative position along the moisture gradient described above for the three species. Fragments of leaves with mature sporangia were collected between April and May 2005. Spore release was promoted by drying the leaf fragments on sheets of smooth paper for 1 week in the laboratory. Spores from the 15 individuals of each species were pooled prior to beginning the culture experiment.

### Experimental treatments

Spores were sown on sterilized mineral agar (see Dyer 1979, p. 282) in 5.5 cm-diameter Petri dishes. The dishes were sealed with Parafilm (American National Can, Chicago, IL, USA) and incubated in a growth chamber (20 °C, PAR 50 µmol m^−2^ s^−1^, 16/8 h photoperiod). Five weeks after sowing, the resulting gametophytes, still asexual at that time, were transplanted to square Petri dishes divided into 25 isolated square cells of 2 × 2 cm each (Bibby Sterilin; Barloworld Scientific, Stone, Staffordshire, UK). Into each cell, one gametophyte was transplanted on 3 cm^3^ of commercial potting soil (‘Blumenerde’, Floragard, Oldenburg, Germany). Three moisture levels were established by watering weekly with 100, 200 or 500 µL of distilled water per cell (hereafter low, medium and high moisture, respectively). These moisture levels were empirically chosen to obtain completely-dry, intermediate and water-saturated soil, respectively, after one week under culture conditions. A total of 200 gametophytes (i.e. eight square dishes) were cultured per species and moisture level, for a total of 1800 gametophytes. Temperature and light conditions were the same as for gametophyte production. Square dishes were rotated weekly within the growth chamber to avoid position effects.

Gametophytes were sampled 13, 18 and 23 weeks after spore sowing. At each time, 56 gametophytes in each species–moisture level combination were harvested. Gametophytes were stained with a solution of acetocarmine-chloral hydrate (Edwards and Miller 1972), heated in a water bath at 50 °C and rinsed with distilled water. They were mounted on microscope slides for observation under the light microscope. For each gametophyte, four variables were recorded: mortality (dead or alive); gender (asexual, female, male or bisexual); automixis (sporophytes present or sporophytes absent) and gametophyte size. For the latter variable, images of mounted gametophytes were obtained with a high-resolution scanner (0.008 mm pixel^−1^) and their area was measured with the program ImageJ (Abramoff *et al*. 2004). Given that gametophytes were grown in isolation, the only possible mating system was automixis (i.e. intragametophytic selfing) and only bisexual gametophytes could form sporophytes. Thus, automixis rate was the percentage of bisexual gametophytes bearing sporophytes. Although no apogamous sporophytes were observed in a previous study of these species (González 2008), sexual origin of sporophytes was checked. Apogamous sporophytes outgrowth from vegetative cells of the gametophyte, instead of from archegonia, and usually show some distinctive traits: multicellular hairs or scales in the embryonic area, tracheids within the cushion, etc. (Huang *et al*. 2011, and references therein).

In order to evaluate antheridiogen production and sensitivity, two kinds of cultures were set up. In both cases, growth conditions were the same as described above. To test each species for the production of antheridiogen, several female gametophytes (antheridiogen source) were located in the centre of the plate, and spores from the same population (antheridiogen target) were sown around them. Thus, source and target gametophytes belonged to the same species. To check whether one species was sensitive to the antheridiogen of the other two species, spores sown around the central female gametophytes were of a different species. Thus, source and target gametophytes belonged to different species in each plate. Four replicates were made for each combination. Three months after sowing, 40 gametophytes were randomly sampled around the antheridiogen source from the four replicates of each combination. Gametophytes were fixed in the same way as commented above, and mounted on microscope slides for gender examination.

### Statistical analysis

Generalized linear models (GLMs; McCullagh and Nelder 1989) were used to analyse the gametophyte variables (mortality, gender, size and automixis), using the GENMOD procedure of SAS 9.0 (SAS Institute 2002). Generalized linear models were chosen because of unbalanced sample sizes, empty cells and variables departing from a normal distribution. The error distributions for mortality, gender, size and automixis were binomial, multinomial, Poisson and binomial, respectively, and the link functions were logit, cumulative logit, log and logit, also respectively, because under these conditions the explained variation was maximal (Guisan *et al*. 2002). The explanatory variables considered in the models were species and moisture level. In addition, gender was included as an effect in the size model, and harvest time in the gender and size models. All of these variables were considered as fixed effects. In those models with no significant interactions among variables, species means and moisture levels means were pairwise compared using LSMeans option (*P* < 0.05) of GENMOD.

## Results

### Mortality

**Figure 1.**
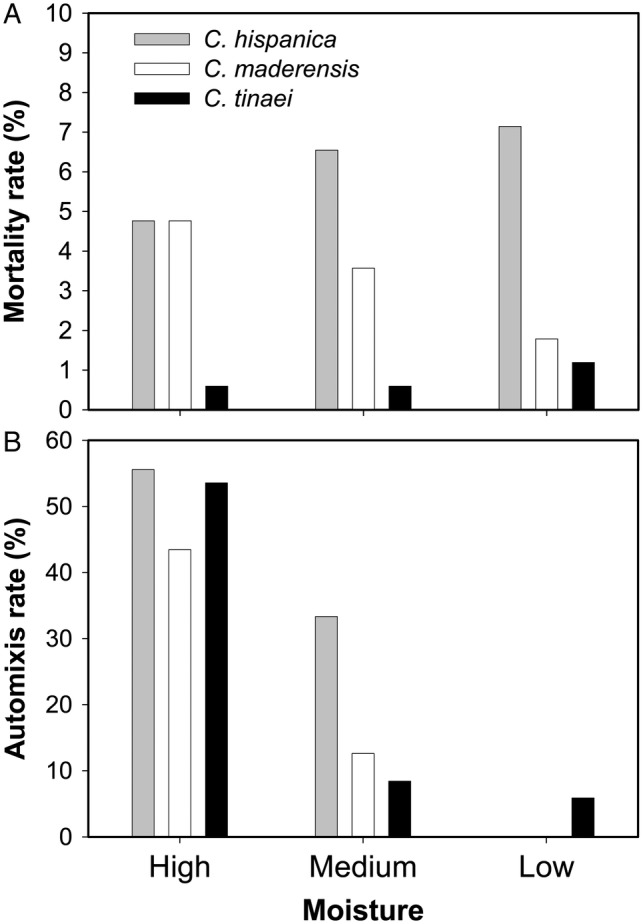
Mortality (A) and automixis (B) of the three *Cheilanthes* species under the three moisture levels. Gametophytes were maintained in isolation and thus automixis was the only possible breeding system. Automixis rate was the percentage of gametophytes bearing sporophytes based only on bisexual gametophytes.

Gametophytes of the three *Cheilanthes* species exhibited successful vegetative and reproductive development overall. Mortality was low and did not differ significantly among the three moisture levels, but it did among species (Table 1). After pooling moisture levels, the three species means were significantly different (LSMeans pairwise comparison) and decreased in the order: *C. hispanica* > *C. maderensis* > *C. tinaei* (Fig. 1A).

**Table 1.** Significance tests for the differences of gametophyte variables among the three *Cheilanthes* species under three moisture levels. In the models for gender and size, the effects of the three harvest times (13, 18 and 23 weeks since sowing) were also included. In addition, gender was considered as an effect in the size model. The error distributions for mortality, gender, size and automixis were binomial, multinomial, Poisson and binomial, respectively (GENMOD procedure in SAS). The automixis model was based on the subsample of bisexual gametophytes. Significant values are in bold.

Dependent variable	Effect in model	Significance test		
		df	*χ*^2^	*P*
Mortality	Species	2	24.11	**<0**.**0001**
	Moisture	2	0.01	0.9966
	Species × moisture	4	3.82	0.4303
Gender	Species	2	946.19	**<0**.**0001**
	Moisture	2	437.71	**<0**.**0001**
	Time	2	328.89	**<0**.**0001**
	Species × moisture	4	36.90	**<0**.**0001**
	Species × time	4	20.42	**0**.**0004**
	Moisture × time	4	1.81	0.7699
	Species × moisture × time	8	13.08	0.1091
Size	Species	2	78.18	**<0**.**0001**
	Moisture	2	92.27	**<0**.**0001**
	Time	2	35.63	**<0**.**0001**
	Gender	2	5.64	0.0597
	Species × moisture × time × gender	49	177.47	**<0**.**0001**
Automixis	Species	2	4.00	0.1350
	Moisture	2	36.09	**<0**.**0001**
	Species × moisture	3	4.97	0.1743

### Gender

**Figure 2.**
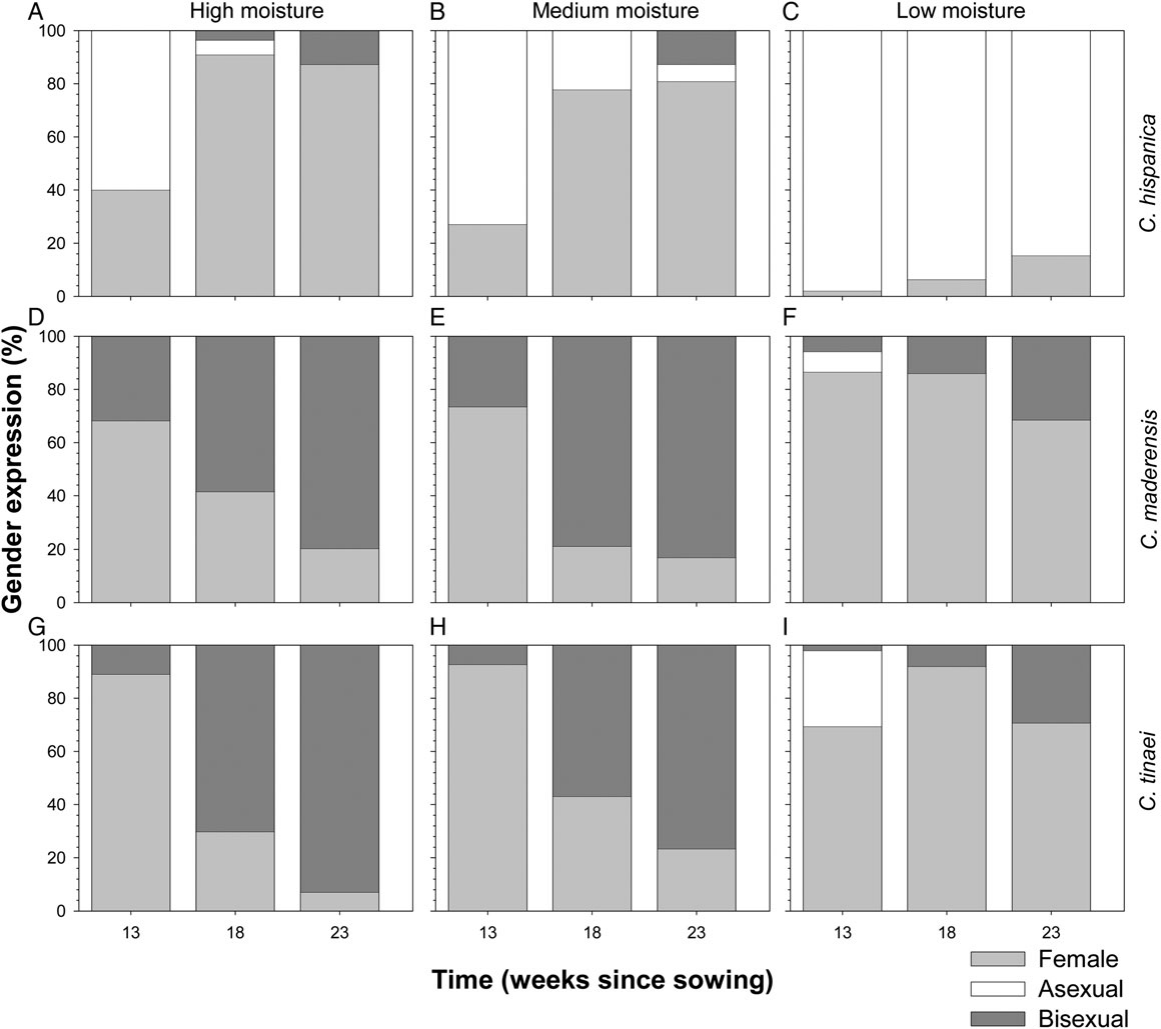
Gender expression of the three *Cheilanthes* species under the three moisture levels. (A–C) *Cheilanthes hispanica* at high (A), medium (B) or low (C) moisture; (D–F) *C. maderensis* at high (D), medium (E) or low (F) moisture; (G–I) *C. tinaei* at high (G), medium (H) or low (I) moisture.

Gender in the different watering treatments experiment was significantly affected by the factors: species, moisture level and harvest time, and by the species × moisture and species × time interactions (Table 1). The three species followed the same gender expression in all moisture levels, from asexual to female to bisexual (Fig. 2), with the exception of *C. hispanica* in the low-moisture level, which passed from asexual to female but not to bisexual (Fig. 2C). None of the gametophytes became male in this moisture gradient experiment. Both the diploid *C. maderensis* and the tetraploid *C. tinaei* had greater percentages of female or bisexual gametophytes than the diploid *C. hispanica* in all times and moisture levels, except in the high-moisture level 23 weeks after sowing, when all gametophytes of the three species were sexually mature (Fig. 2A, D and G). The abundance of bisexuals decreased with decreasing moisture levels in all three species.

### Size

**Figure 3.**
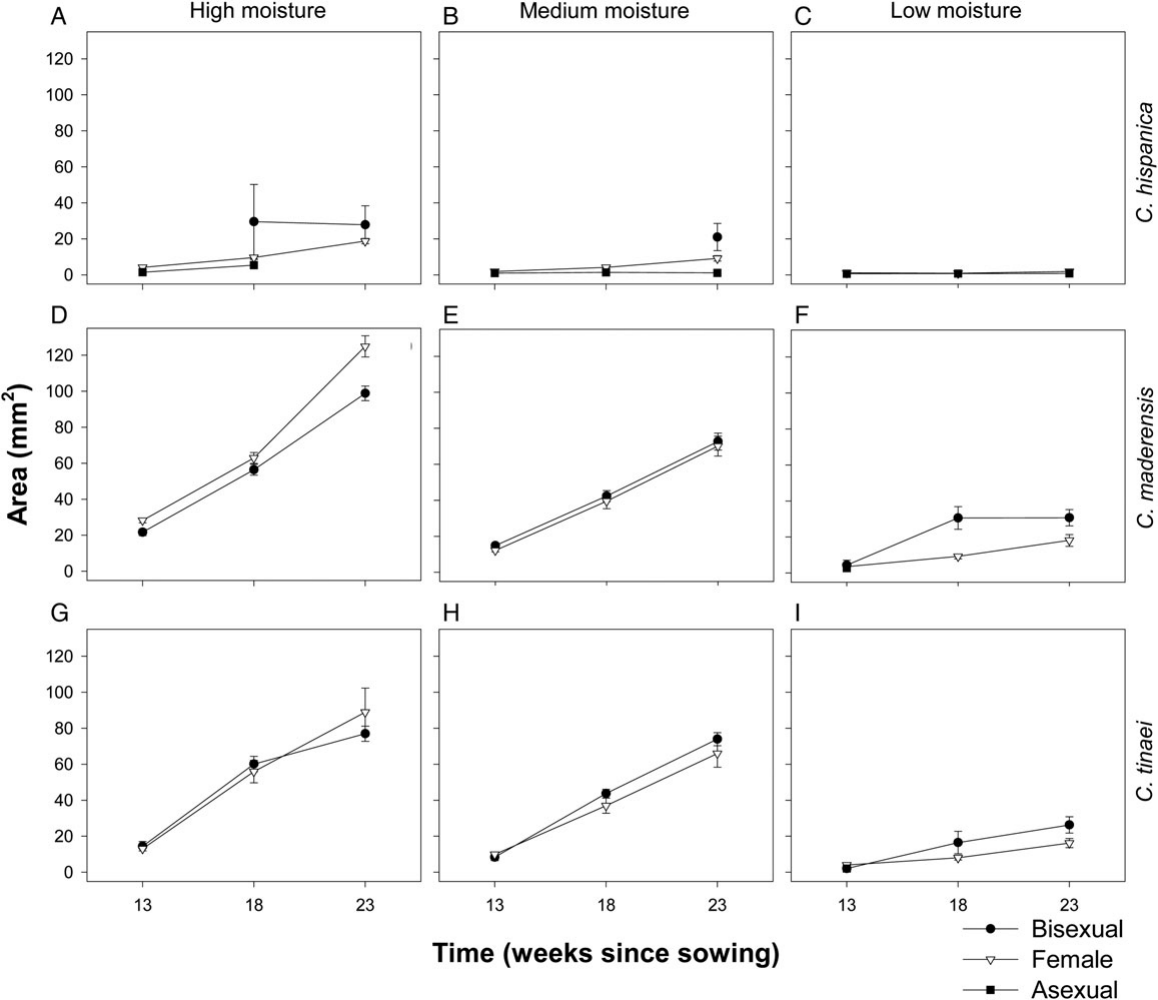
Size (mean ± SE) of the three *Cheilanthes* species under the three moisture levels. (A–C) *Cheilanthes hispanica* at high (A), medium (B) or low (C) moisture; (D–F) *C. maderensis* at high (D), medium (E) or low (F) moisture; (G–I) *C. tinaei* at high (G), medium (H) or low (I) moisture.

Size was affected by the species, moisture and time, but not by gender (Table 1). The four-way interaction of all main factors was also significant. For each species in each moisture level and time, gametophytes of different genders were generally of similar size (Fig. 3). In all times and moisture levels, gametophytes of *C. hispanica* were smaller than those of *C. maderensis* and *C. tinaei*, which in turn had similar sizes. In all three species, gametophytes decreased in size with decreasing moisture levels (Fig. 3).

### Automixis

Automixis of bisexual gametophytes was affected by moisture but not by species (Table 1). The percentage of bisexual gametophytes bearing sporophytes decreased from ∼50 % at high moisture to <10 % at low moisture (Fig. 1B). The three moisture levels means differed significantly (LSMeans test) from one another. Based on morphological traits, all sporophytes found were sexually formed, i.e. no apogamous sporophytes were observed.

### Antheridiogen

**Figure 4.**
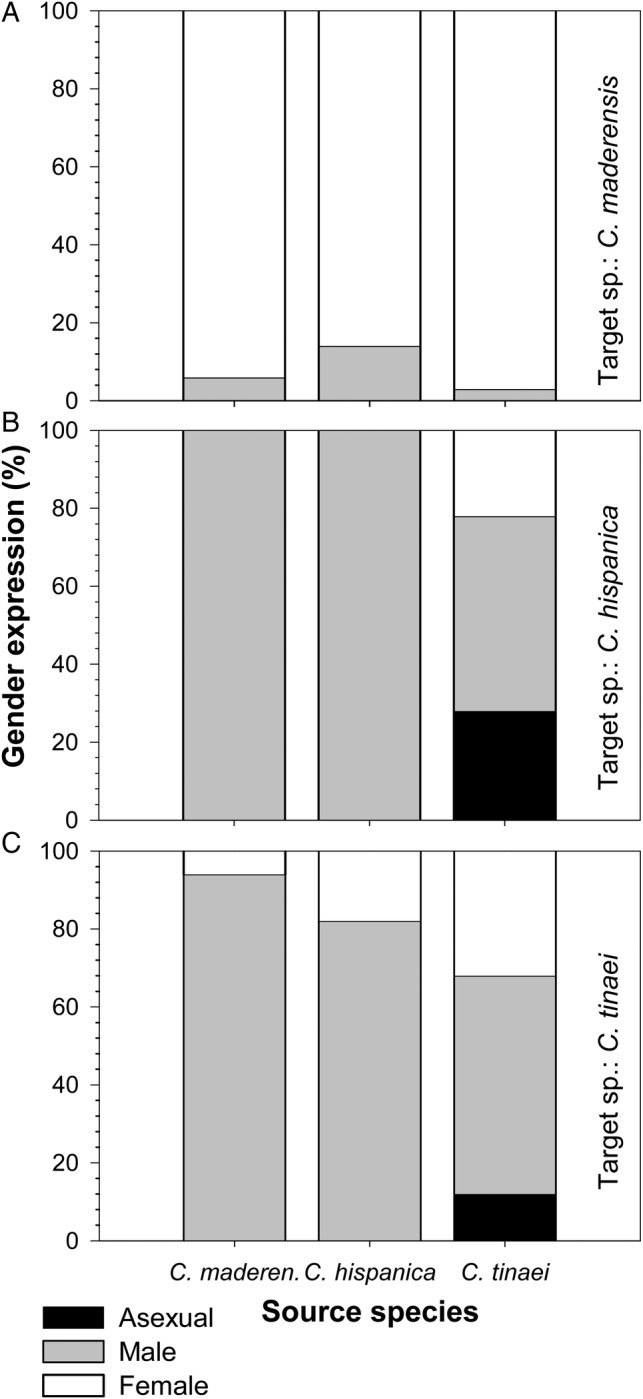
Gender expression after 3 months of culture of gametophytes grown around adult gametophytes of the three *Cheilanthes* species as antheridiogen sources: (A) *C. maderensis* as target species; (B) *C. hispanica* as target species and (C) *C. tinaei* as target species.

Female gametophytes of the three species produced antheridiogens that favoured maleness both within and among species but this response varied depending on the source–target species combination (Fig. 4). *Cheilanthes maderensis* showed the weakest response to its own antheridiogen and to that of the other two species (Fig. 4A). The higher percentage of males, 14 %, was produced with *C. hispanica* gametophytes as the antheridiogen source. *Cheilanthes hispanica* was very sensitive to its own antheridiogen and to the one of *C. maderensis* (Fig. 4B). In both cases, 100 % of male gametophytes were produced. This percentage decreased to 50 % when the source of antheridiogen was *C. tinaei*. The reaction of *C. tinaei* was high for the antheridiogen of *C. maderensis* and of *C. hispanica*, 82 and 94 % of males were produced, respectively (Fig. 4C). With its own antheridiogen, the percentage of males decreased to 56 %, and some gametophytes, 12 %, remained vegetative. In summary, *C. maderensis* exhibited little sensitivity to conspecific antheridiogen, *C. tinaei*, a moderate response and *C. hispanica*, a very strong response.

## Discussion

### Environmental sex determination in *Cheilanthes*

In some fern species, harsh growth conditions, such as poor substrate or high density, lead to small, male gametophytes (DeSoto *et al*. 2008, and references therein). In contrast, gametophytes of this diploid-allotetraploid *Cheilanthes* complex do not exhibit an increase in maleness related to an increase in harsh environmental conditions, in our case water availability. Instead, in isolated conditions, the sexual ontogeny of gametophytes from asexual to female to bisexual is present and does not depend upon the degree of soil moisture. Interestingly, different watering regimes in our experiments affected gametophyte growth, with bigger gametophytes under moister conditions, but this difference in sizes did not translate into different sex expressions. Specifically, small gametophyte sizes were not coupled with the production of antheridia as observed in other ferns such as *Woodwardia radicans* (Quintanilla *et al*. 2007; DeSoto *et al*. 2008). In our experiment, however, water stress affected the proportion of females and bisexuals, with lower moisture levels translating into fewer bisexuals (Fig. 2), which suggests that there is some ESD in this *Cheilanthes* species. Moreover, in this species group, it is clear the presence of an antheridiogen promotes maleness, as is the case in many homosporous ferns (Schneller 2008, and references therein). Many studies (e.g. Testo *et al*. 2015) include as controls *Onoclea sensibilis*, the most sensitive species to antheridiogens, and *Pteridium aquilinum*, the antheridiogen of which induces responses in a wide spectrum of species. These controls can be useful to verify that a study species actually does not produce antheridiogens and does not respond to it, respectively. However, our three study species produced antheridiogens that in turn induced maleness in the three study species and thus these controls are not necessary.

Some species, particularly in fish, are under GSD but sex ratios can be further modified by environmental factors (Ospina-Álvarez and Piferrer 2008; Baroiller *et al*. 2009). Dependence solely upon environmental factors can expose species to biased sex ratios, with potentially harmful effects for populations (Stelkens and Wedekind 2010). For example, sexual reproduction would not be possible if all individuals were male under harsh conditions. However, as seen above, water scarcity does not favour maleness in the study species.

In ESD, sex is determined by the interaction between environmental, genetic and hormonal factors (Valenzuela *et al*. 2003; Stelkens and Wedekind 2010). This may be what we actually see in homosporous ferns: they have the tendency to follow a default development programme, but it can vary depending on environmental signals (Masuyama 1975; Rubin and Paolillo 1983; Korpelainen 1994). The sequence of gender expression from asexual to female to bisexual, as we found in *Cheilanthes*, is considered the default sexual ontogeny in ferns (Sánchez Vilas and Pannell 2012), and has been traditionally explained as a mechanism to assure fertilization by inbreeding (Lloyd 1974). The lower percentage of bisexuals we found at lower levels of water availability may be related to the reduction of vegetative growth. Moisture deficit produced smaller gametophytes (Fig. 3). Because a multicellular meristem and a multilayered cushion is necessary for the production of bisexual gametophytes, the low proportion of bisexuals at lower moisture levels is probably due to not reaching the size and morphology thresholds required due to the delayed vegetative development under those conditions. Our results are not in concordance with the rise in reproductive effort (i.e. gametangia to size ratio) at low growth rates observed in other species that increase their allocation to male reproduction instead of vegetative growth (Greer and McCarthy 1999).

### Differences between ploidy levels

The allotetraploid *C. tinaei* showed gender expression and growth reduction in response to water scarcity more similar to those of the parent *C. maderensis*. Both species had higher percentages of females and bisexuals than *C. hispanica*, which, in turn, showed more growth reduction. In contrast, gametophyte size of an allotetraploid *Dryopteris* was intermediate relative to its parents (Jiménez *et al*. 2008). Regarding mortality, it was equally low in all the three moisture regimes for each species. Low mortality is not surprising as fern gametophytes exhibit desiccation tolerance that is greater in species from xeric habitats (Watkins *et al*. 2007). However, there were significant differences in mortality among species, with *C. tinaei*, exhibiting lower percentages of mortality than both diploids. This result suggests hybrid vigour (but see below).

Polyploids of flowering plants tend to have higher selfing rates than their diploid relatives (Stebbins 1950; Mable 2004; Barringer 2007), and this trend has been observed in homosporous ferns too (Soltis and Soltis 1987; Masuyama and Watano 1990). Against this expectation, *C. tinaei* did not develop significantly more automictic sporophytes than its diploid parents did across treatments (Fig. 1B). This contrasts with the expectation for polyploid ferns to exhibit less inbreeding depression than their diploid parents and, therefore, being able to tolerate higher selfing rates (Soltis and Soltis 2000). In addition, it is generally assumed that polyploids are more successful than their diploid parents, at least in novel and disturbed habitats (Levin 1983; Otto and Whitton 2000). This is not valid for the study species as, in the Iberian Peninsula (Anthos 2013), the diploid *C. maderensis* occupies a wider area than the other two species, while the other diploid, *C. hispanica*, occupies the smallest one. This could mean that the supposedly superior colonizing ability of the tetraploid is surpassed by that of one of the diploid parents. In other terms, that there is not hybrid vigour that gives advantage to the tetraploid relative to its parents, and the allotetraploid exhibits an intermediate colonizing ability between both diploids. Evidence of very limited advantage of allopolyploids has been found in North American allotetraploid species of *Dryopteris* (Sessa and Givnish 2014). An intermediate behaviour in other traits of an allotetraploid relative to its diploid parents was observed in some *Asplenium* (Prada *et al*. 1995) and *Polystichum* species (Pangua *et al*. 2003). The response to the antheridiogen showed a similar model, high effect in one diploid, *C. hispanica*, very low in the other, *C. maderensis*, and intermediate, relative to both diploids, in the allotetraploid, *C. tinaei*.

### Adaptation to the habitat

The ability of gametophytes to generate young sporophytes through automixis was positively correlated with moisture, as expected from their need for water for fertilization. The three species under study can self-fertilize and raise completely homozygous sporophytes, which represents an advantage for long distance dispersal, but a disadvantage for genetic variation over the long-term. It may also be an adaptation to xeric habitats, where microsites for spore germination are scarce and sperm movement between gametophytes may be hindered by the absence of water. We did not find aborted embryos and selfed sporophytes showed normal growth. However, it must be noted that we only studied early development of sporophytes (a 23 weeks culture) whereas inbreeding depression might act later in the life cycle (e.g. Morgante *et al*. 1993).

Moreover, we found that the three study species are sensitive to the action of antheridiogen. This fact allows us to advance the following scenario. Adequate water availability would allow germination of several spores present in a safe site. The fastest gametophytes to develop would become female and secrete antheridiogen, thus inducing maleness in the surrounding ones and promoting outcrossing. In harsh conditions, however, only a few isolated spores would germinate, and their final development as bisexuals would facilitate self-fertilization assuring reproduction as observed in some flowering plants (Evans *et al*. 2011). This mixed mating system, known in fern species with high colonization potential (Wubs *et al*. 2010), would result from a combination of a partial ESD (i.e. response to antheridiogen) and, as commented above, a pre-set sexual ontogeny from asexual to female to bisexual. These strategies would confer, respectively, two advantages: promotion of outcrossing and reproductive assurance (Goodwillie *et al*. 2005). It could be predicted that, as the response to antheridiogens is weaker in *C. maderensis*, populations of this species would show higher selfing rates than populations of *C. tinaei* and *C. hispanica*.

## Conclusions

In the allopolyploid complex examined here, the effect of water availability on gender expression is small. Isolated gametophytes show an asexual to female to bisexual sequence that does not depend upon the degree of soil moisture. Both gender expression and growth reduction in response to water scarcity of the allotetraploid resemble one of the diploid parents. In all moisture regimes, mortality is lower in the allotetraploid, suggesting hybrid vigour, whereas automixis rate is similar in the three species. Sex is environmentally determined by antheridiogens. The allotetraploid shows an intermediate response to antheridiogens relative to its parents. The production of bisexual gametophytes allows for self-fertilization, while the presence of male gametophytes when antheridiogen is present promotes outcrossing. Thus, it can be speculated that this mixed mating system could be advantageous in the xeric habitat of these species. The study of population's genetic structure and of ecological niche differences among the three species would help to clarify their reproductive behaviour and the reasons behind their geographical distribution.

## Sources of Funding

This work was supported by the Ministerio de Ciencia e Innovación of the Spanish Government, project number CGL2010-20124.

## Contributions by the Authors

S.P., E.P. and L.G.Q. conceived and designed the experiments; S.P. and E.P. carried out the experiments and collected the data; L.G.Q. ran the statistics; all four authors collaborated on the discussion and writing of this article.

## Conflict of Interest Statement

None declared.
